# Video feedback parent‐infant intervention for mothers experiencing enduring difficulties in managing emotions and relationships: A randomised controlled feasibility trial

**DOI:** 10.1111/bjc.12388

**Published:** 2022-08-26

**Authors:** Kirsten Barnicot, Morgan Welsh, Sarah Kalwarowsky, Eloise Stevens, Jane Iles, Jennie Parker, Maddalena Miele, Tara Lawn, Laura O'Hanlon, Sushma Sundaresh, Ola Ajala, Paul Bassett, Christina Jones, Paul Ramchandani, Mike Crawford

**Affiliations:** ^1^ Research and Development/Perinatal Mental Health Service Central & North West London NHS Foundation Trust London UK; ^2^ Division of Psychiatry Imperial College London London UK; ^3^ Centre for Mental Health Research City University of London London UK; ^4^ Department of Psychology University of Surrey Guildford UK; ^5^ Perinatal Mental Health Service East London NHS Foundation Trust London UK; ^6^ Perinatal Mental Health Service Oxleas NHS Foundation Trust London UK; ^7^ Perinatal Mental Health Service Camden & Islington NHS Foundation Trust London UK; ^8^ Statsconsultancy Ltd Amersham UK; ^9^ Faculty of Education University of Cambridge Cambridge UK

**Keywords:** infant mental health, parent–infant intervention, perinatal mental health, randomised controlled trial

## Abstract

**Objectives:**

Parents experiencing mental health difficulties consistent with “personality disorder”, often related to a history of complex trauma, may face increased challenges in parent–child relationships and child socioemotional development. There are no published randomised controlled trials (RCTs) evaluating perinatal parent–child interventions for this population. We evaluated the feasibility and acceptability of undertaking an RCT of the video feedback intervention for positive parenting adapted for perinatal mental health (VIPP‐PMH).

**Design:**

Feasibility study incorporating a pilot RCT.

**Methods:**

Mothers with enduring difficulties in managing emotions and relationships, consistent with a “personality disorder”, and their 6‐ to 36‐month old infants were randomly allocated to receive six sessions of VIPP‐PMH (*n* = 20) or usual care alone (*n* = 14).

**Results:**

76% of eligible mothers consented to participate. Intervention uptake and completion rates were 95% (≥1 VIPP‐PMH session) and 70% (6 sessions), respectively. Follow‐up rates were 85% at month 5 and 65% at month 8 post‐baseline. Blinded observer‐ratings of maternal sensitivity in parent–child interaction favoured the intervention group at month 5 (RR = 1.94, 95% CI 0.67–5.63) and month 8 (RR = 1.91, 95% CI 0.68–5.33). Small changes over time in self‐rated parenting confidence and stress favoured the intervention group. There were no clear intervention effects on maternal non‐intrusiveness or mental health, or on child behaviour problems, emotional functioning, or self‐regulation.

**Conclusions:**

An RCT of VIPP‐PMH is feasible and acceptable to implement with mothers experiencing difficulties consistent with perinatal “personality disorder”. A fully powered definitive RCT should be undertaken.

**Practitioner Points**

- The video feedback intervention for positive parenting adapted for perinatal mental health(VIPP‐PMH) is feasible and acceptable to implement with women experiencing enduring difficulties in managing emotions and relationships consistent with a personality disorder
- VIPP‐PMH has potential benefits for reinforcing or improving maternal sensitivity in parent–child interaction and improving parenting confidence and stress in this population.
- The findings underscore the value of early intervention focussed on supporting positive parent–child relationships.

## BACKGROUND

Parents who experience enduring difficulties in managing their emotions and interpersonal relationships, consistent with a “personality disorder”, are often highly motivated to provide a positive parenting experience for their children (Bartsch et al., 2016, Dunn et al., 2020). Yet, they can sometimes feel negative about themselves as parents and their relationship with their child and can struggle to feel confident and competent (Bartsch et al., 2016; Dunn et al., 2020; Zalewski et al., 2015). As the location of mental health difficulties within the core self or “personality” can be considered stigmatising, but an alternative conceptualisation has not been agreed (Consensus Statement Group, 2018; Watts, 2019), we will henceforth place this terminology in quotes. It is also important to acknowledge the aetiological significance of childhood adversity and trauma (Porter et al., 2020; Steele et al., 2019) and the strong conceptual and empirical overlap between “borderline personality disorder” and complex post‐traumatic stress disorder (Cloitre et al., 2014; Driessen et al., 2002; Frost et al., 2020; Giourou et al., 2018; Jowett et al., 2020).

Some parents experiencing difficulties consistent with “personality disorder” can struggle to accurately tune into and respond sensitively to their child's cues and may be intrusive in the interaction (Conroy et al., 2012; Crandell et al., 2003; Elliot et al., 2014; Hobson et al., 2005; Johnson et al., 2006; Kiel et al., 2011; Newman & Stevenson, 2005; White et al., 2011). These difficulties in parent–child interaction increase the risk of insecure and disorganised parent–child attachment and of child socioemotional difficulties (De Wolff & van Ijzendoorn, 1997; Fearon et al., 2010; van Ijzendoorn et al., 1999). Relatedly, children who have a parent meeting diagnostic criteria for a “personality disorder” are more likely to experience insecure or disorganised attachment in infancy (Crandell et al., 2003; Hobson et al., 2005), emotional and behavioural dysregulation in toddlerhood (Conroy et al., 2012), and mental health difficulties in early and middle childhood (Abela et al., 2005; Barnow et al., 2006; Berg‐Nielsen & Wichström, 2012; Herr et al., 2008; Macfie & Swan, 2009; Weiss et al., 1996).

Existing research has arguably overly focussed on parenting difficulties, neglecting to explore parenting strengths and resilience (Petfield et al., 2015). Yet, all of the reviewed studies identify only an increased probability, not an inevitability, that parents with “personality disorder” and their children will face these challenges. An increased focus on protective factors and resilience is merited.

Given that the first three years of life are a sensitive period of neuroplasticity (Shonkoff & Philips, 2000; Siegel, 2015), research and policy stress the importance of early intervention, through provision of parent–infant interventions for parents experiencing perinatal mental illness (Department of Health, 2012; Heckman, 2008; Heckman & Masterov, 2007; National Health Service, 2019; UK Parliament, 2013). To date, there have been no published randomised controlled trials (RCTs) evaluating parent–child interventions in the perinatal period, for parents experiencing difficulties consistent with “personality disorder”. Parental sensitive responsiveness to their infants may be a particularly important target for intervention to support positive development throughout childhood (Kok et al., 2013). Meta‐analysis has shown that the most effective interventions for supporting sensitivity incorporate feedback to the parent based on videos of parent–infant interaction (Bakermans‐Kranenburg et al., 2003; Dunst & Kassow, 2004; O'Hara et al., 2019). The video feedback intervention for positive parenting (VIPP) has been tested in numerous RCTs in other at‐risk groups, including mothers with bulimia nervosa, insecure attachment or low sensitivity, and children with behavioural problems, and has demonstrated effectiveness in improving parental sensitivity, increasing parent–child attachment security, and reducing child behaviour problems (Juffer et al., 2017; O'Farrelly et al., 2021).

We aimed to undertake an important first stage in evaluating the helpfulness of this intervention for parents experiencing difficulties consistent with a “personality disorder” by adapting the intervention to increase its acceptability and by evaluating the feasibility and acceptability of undertaking a randomised controlled trial.

## METHODS

### Design

A two‐phase feasibility study in which the intervention was piloted using a case series design and modified (Phase 1), followed by a two‐arm parallel pilot randomised controlled trial (Phase 2).

### Protocol

The trial protocol was registered prospectively in the ISRCTN registry: https://www.isrctn.com/ISRCTN10052006.

### Ethical approval

The study was approved by the UK NHS Health Research Authority and granted a favourable opinion by the London—Camden and Kings Cross NHS Research Ethics Committee in June 2017 (17/L0/0669).

### Inclusion criteria

The study included men and women who

- Were experiencing enduring difficulties in managing emotions and relationships consistent with DSM‐V “personality disorder” as assessed using the SCID‐PD Personality Disorder Interview;
- Had parental responsibility for a biological or adopted child aged 6–36 months old at the point of randomisation, with unsupervised contact for at least 24 hours a week;
- Were capable and willing to give informed consent;
- Were aged 16–65 years old;
- Were accessing secondary care mental health services in a participating Trust at the point of study entry.

### Exclusion criteria

The study excluded families in which

- A sibling or co‐parent had already participated in the study.
- The eligible child had a diagnosed learning difficulty, developmental disorder or sensory impairment.
- The eligible parent had English language or learning difficulties of sufficient severity to prevent them completing study measures even with assistance.

### Intervention

The intervention comprised six 90‐minute sessions using the Video‐feedback Intervention to promote Positive Parenting adapted for perinatal mental health (VIPP‐PMH), with a Sensitive Discipline (SD) component included for children aged 11 months or older, delivered approximately every two weeks. Sessions were primarily delivered in participant homes, but clinicians and participants could request clinic‐based delivery if desired. Clinicians delivering the intervention attended a 5‐day accredited training in the VIPP‐SD programme and the VIPP‐PMH adaptations, with supervision by an accredited supervisor prior to each delivered session. Sessions were audio‐recorded with parental consent, and a randomly selected 10% of audio recordings were coded for fidelity by an independent researcher using a VIPP‐SD adherence scale adapted for VIPP‐PMH (O'Farrelly et al., 2018, 2021). Scores range from 1 (did not follow the manual at all) to 5 (followed the manual very closely), with all coded sessions rated at least 3 (“acceptable fidelity”).

In each session, clinicians videoed the parent and child engaging in play and in everyday activities such as mealtime, with developmentally appropriate toys provided by the clinician (Juffer et al., 2015; VIPP Training and Research Centre, 2015). For families receiving the SD component, limit‐setting tasks such as tidying up were also filmed, and tips on sensitive limit‐setting were given. The clinician then watched the videos back with the parent in the subsequent session and gave feedback on the child's interactive and play behaviour from the perspective of the child, highlighting the child's attachment and exploratory behaviours and reinforcing parents' sensitive responses. When viewing moments where a parent responded less sensitively or missed the child's cue, the clinician gave tips for alternative ways of responding and flagged moments of more optimal parental responsiveness elsewhere in the interaction.

Based on pre‐study feedback from our lived experience co‐researchers and parent advisory group, the intervention was adapted before study initiation for parents experiencing perinatal mental health difficulties consistent with “personality disorder” by adding additional material around managing self‐critical feelings and anxieties about being judged by the clinician. At the start of each session, the non‐judgemental nature of the intervention and the focus on the child was emphasised. Parents were asked to notice any self‐critical thoughts or worries about being judged and put them aside to re‐focus their attention on their child. Each session ended with a debrief where any difficult thoughts and feelings were explored. Following feedback from participants receiving the intervention during the Phase 1 case series, the intervention was further modified to include a brief discussion of the parent's hopes and expectations and alignment of the parent's goals with the aims of VIPP‐PMH. For example, if the parent's goal was to change the way they feel about their child, the clinician would explain that whilst the intervention will not directly focus on the parent's feelings, they will be help understand and respond to their child in ways that may promote more positive feelings between them.

### Control condition

Both intervention and control participants received care‐as‐usual from perinatal, adult mental health, primary care, and/or social services. Additionally, all participants received booklets on early child development, coping with crying and positive parenting (Department of Education, 2013, National Society for the Prevention of Cruelty to Children, 2013a, 2013b).

### Recruitment

Participants were recruited from community perinatal mental health and personality disorder services in four NHS Trusts in London, United Kingdom, between November 2017 and June 2019. Clinicians in participating services identified potentially eligible participants who had a confirmed personality disorder diagnosis, and/or who scored over 8 on the Standardized Assessment of Severity of Personality Disorder screening measure (SAS‐PD, Olajide et al., 2017), and/or were deemed by the clinical team to be experiencing enduring difficulties in managing emotions and relationships consistent with a “personality disorder”, based on a comprehensive clinical assessment by an experienced clinician including an evaluation of psychiatric status, past psychiatric history, past and current level of function in relationship, employment and work domains, and cross‐referencing with medical and mental health electronic records. Following a verbal explanation of the research project by the clinician, interested participants were asked to give verbal consent for researcher contact. A researcher then met with the participant to provide further written information and record written informed consent where the participant was willing to confirm eligibility using the SCID‐PD Personality Disorder Interview and administer baseline measures.

### Phase 1 Case series

The intervention and study measures were piloted with a case series of nine parents who met the inclusion criteria, followed by qualitative feedback interviews with parents and with the clinicians delivering the intervention.

### Phase 2 Pilot randomised controlled trial

Individual participants were randomly allocated to the intervention or control condition using a 1:1 allocation ratio. An independent researcher based at Imperial College London used the Sealed Envelope website (Sealed Envelope Ltd, 2022) to generate an allocation list in blocks of 6, stratified by NHS Trust. Allocation was concealed from the participant and all members of the clinical and research team until the point of randomisation. Following informed consent and the baseline assessment, the independent researcher randomised the participant and notified the research team.

#### Sample size

Following the National Institute for Health Research Design Service London recommendations (Hooper, 2013), we aimed to recruit 40 participants to enable detection of study consent and completion rates of 50%, with 95% confidence intervals of ±11% and ±15%, respectively.

#### Baseline measures

At baseline, the following information was collected to characterise the sample:

- Sociodemographic factors. Age, gender identity, ethnicity, target child age and gender assignment, number of children, employment status, and relationship status.
- The Structured Clinical Interview for DSM‐5 Personality Disorders and the Standardized Assessment of Severity of Personality Disorder to confirm eligibility and characterise personality difficulties.
- The Trauma History Questionnaire and the International Trauma Questionnaire (Disturbances in Self‐Organisation subscale; Cloitre & Bisson, 2013; Cloitre et al., 2018; Dokkedahl et al., 2015) to assess trauma and complex PTSD symptoms.
- The Edinburgh Postnatal Depression Scale (Cox et al., 1987) to assess depressive symptoms.
- Primary diagnosis was recorded by clinical staff from participants' medical records.
- Service Use and Risk History. Medication prescription, history of emergency department visits and inpatient hospitalisation for psychiatric reasons, and history of self‐harm were documented using a standardised interview developed for the study (Supporting Information).

#### Primary outcome measure

Percentage of participants in the intervention arm receiving (≥1 session) and completing (≥4 sessions) the intervention, by the time of the 8‐month follow‐up.

#### Secondary outcome measures

- Additional measures of feasibility: percentage of participants deemed potentially eligible by their clinician and given information about the research, consenting to be contacted by the researchers; percentage of eligible participants contacted by the researchers consenting to participate; percentage of participants completing the outcome measures at each follow‐up. Qualitative feedback from participants on intervention feasibility and acceptability is reported elsewhere.

The following data were collected at baseline and the month 5 and 8 follow‐ups:

- Parental Sensitivity and Non‐Intrusiveness. This was assessed using the observer‐rated Emotional Availability Scales, 4^th^ Edition (Biringen, 2008; Biringen et al., 2014) to rate video clips of parents and children playing together. Parental sensitivity was defined as clear perception of, and appropriate responsiveness to, the child's emotional expressions and creation of a generally positive, genuine, and authentic affective climate through congruent verbal and non‐verbal emotional expressions (Biringen et al., 2014). Parental non‐intrusiveness was defined as following the child's lead during the interaction, avoiding over‐direction and interference, and providing support for a level of child autonomy commensurate with their developmental age. Parental sensitivity and non‐intrusiveness were each scored on a scale from 1 to 7. Ratings between 7 and 5.5 are categorised as Sensitive/Non‐intrusive respectively. The researcher assessing this was blind to intervention allocation, was trained by and achieved reliability with the scale developer, and then achieved high inter‐reliability with another trained researcher by independent coding of eight videos from the pilot phase of the study (Weighted Cohen's Kappa *k* = 1.0 and *k* = 0.89 for the sensitivity and non‐intrusiveness subscales respectively).
- Parenting Confidence. This was assessed using the parent‐rated 16‐item Parental Sense of Competence Scale (Johnston & Mash, 1989). Possible scores range between 17 and 102, with higher scores indicating greater parental self‐confidence.
- Parenting Stress. This was assessed using the parent‐rated 18‐item Parental Stress Scale (Berry & Jones, 1995). Possible scores range between 18 and 90, with higher scores indicating greater parental stress.
- Parental Mental Health. This was assessed using the parent‐rated 10‐item Clinical Outcomes in Routine Evaluation (CORE‐10, Evans et al., 2002) assessing subjective well‐being, mental health problems, life functioning, and risk to self/others. Possible scores range between 0 and 4, with higher scores indicating poorer mental health.
- Child Behaviour Problems. This was measured using the parent‐rated 42‐item Brief Infant‐Toddler Social and Emotional Assessment (BITSEA; Briggs‐Gowan & Carter, 2002). Age‐dependent cut‐off scores were used to classify children at risk of behavioural problems. During the pilot phase, the Child Behaviour Checklist (Achenbach, 1992) was also used but was discontinued following feedback from parents that its length and overlap with the BITSEA made it burdensome to complete.
- Child Attachment, Emotional Functioning and Self‐Regulation. This was assessed using the parent‐rated Attachment and Emotional Functioning, and Self‐Regulation subscales of the Infant‐Toddler Symptom Checklist (DeGangi, 1991). Higher scores indicate greater difficulties with attachment and emotional functioning or self‐regulation. Age‐specific versions of this questionnaire were used according to the child's age (6–9 months; 10–12 months; 13–18 months; 19–24 months; 25–44 months), thus taking into account age‐dependent differences in developmentally appropriate child behaviour.
- Information was also collected on health service use in order to understand what “usual care” had been accessed by participants, using a standardised interview developed for the study.

#### Criteria for progression to a definitive trial

These were pre‐specified in our funding application as (1) a minimum of 66% of those in the intervention arm of the trial receive VIPP‐PMH; (2) a consensus by the trial team and our advisory group of PPI representatives and clinicians, that the qualitative feedback from trial participants and interveners indicates that the intervention and a future definitive trial are feasible and acceptable.

### Statistical analysis

Feasibility parameters were calculated as described above. Between‐group differences and within‐group changes in the outcome variables were assessed by comparing the percentage change and calculating risk ratios (RRs) and confidence intervals for dichotomous variables and by comparing mean scores and calculating Hedge's g effect sizes and confidence intervals for continuous variables. Median scores were used for comparison of subscale scores. Where >5% of items on a measure were missing, the participant was dropped from the analysis of that measure. Mean imputation based on remaining item scores was used where 0< ≥ 5% of items were missing. Statistical significance testing was not undertaken as the study was not neither designed nor powered to detect statistically significant differences.

## RESULTS

**FIGURE 1:**
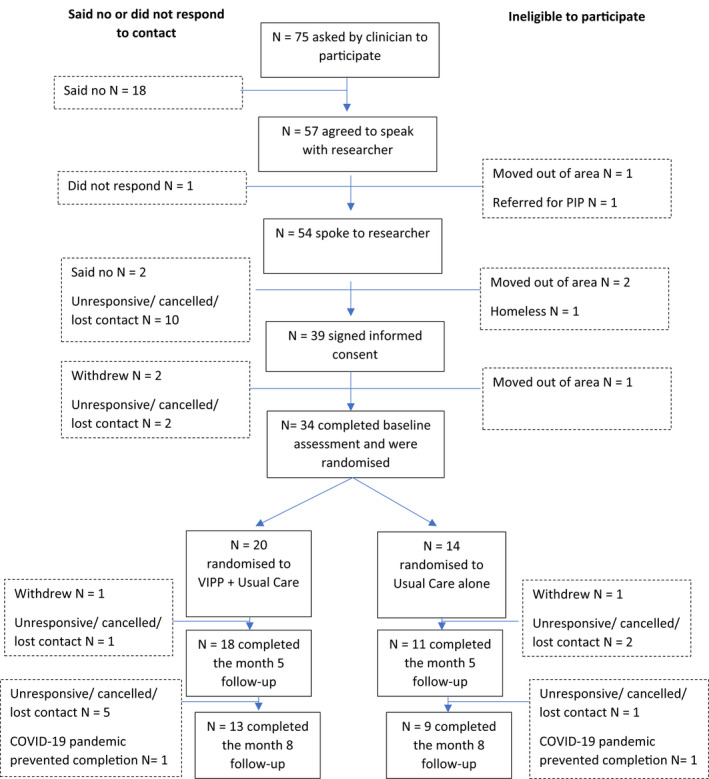
Participant flow through the study. PIP, Parent Infant Psychotherapy

Following the initial VIPP‐PMH case series in nine parents (Phase 1), thirty‐nine parents gave informed consent to participate in the pilot randomised controlled trial (Phase 2) and at this point were given a participant ID, linked to a concealed allocation status. Participant flow through the Phase 2 pilot randomised controlled trial is shown in Figure 1. Allocation status was not revealed until after the baseline assessment was completed. For some parents, there was a gap between informed consent and the baseline assessment as the child was not yet aged 6 months at the time of study entry. Five of these parents withdrew, did not respond to contact, or became ineligible for study participation, before the baseline assessment could be completed, and hence before their allocation status was revealed. Of the 34 parents who completed the baseline assessment and whose allocation status was therefore revealed, twenty were randomly allocated to receive VIPP‐PMH plus usual care and fourteen to receive usual care alone. Care‐as‐usual received primarily included health visitor (62%), perinatal mental health (48%), and general practice (41%) input (Table S1). No serious adverse events or reactions were recorded during the trial.

### Participant characteristics

Participant characteristics are summarised in Table 1. Notably, although the study aimed to include both mothers and fathers, only two fathers were referred to the study and neither had sufficient contact with their children to be eligible for participation. Participants in the present study will henceforth be described as “mothers”.^1^ The sample was ethnically diverse and had high levels of social disadvantage including single parenthood, unemployment, and/or children's social service involvement. The most commonly recorded primary diagnoses were depressive disorders, whilst just over a third had a recorded primary diagnosis of emotionally unstable (borderline) personality disorder on their medical records. During the SCID‐PD interview, most mothers exceeded the diagnostic threshold for borderline “personality disorder”, followed by avoidant, obsessive‐compulsive, and/or paranoid “personality disorders”. Nearly three quarters of the sample had a history of physical and/or sexual trauma, most commonly beginning in childhood, and nearly two thirds exceeded the diagnostic threshold for disturbances in self‐organisation associated with complex PTSD. The sample size precluded statistical testing of baseline differences between mothers in the control and intervention arm. Examination of descriptive differences indicated that mothers in the intervention arm was more likely than mothers in the control arm to be single parents (45% vs. 29%) and had higher Edinburgh Postnatal Depression scale and Complex PTSD Disturbances in Self‐Organisation scores, whereas mothers in the control arm were more likely than mothers in the intervention arm to identify as an ethnic minority (66% vs. 35%) and report a history of self‐harm (100% vs. 70%).

**TABLE 1:** Sample characteristics at baseline assessment

	Intervention (*n* = 20)	Control (*n* = 14)	Total sample (*n* = 34)
	*n* (%)	*n* (%)	*n*(%)
Maternal ethnicity			
White British	9 (45%)	3 (22%)	12 (35%)
White Other	4 (20%)	2 (14%)	6 (17%)
Black British	1 (5%)	2 (14%)	3 (9%)
Mixed	0 (0%)	3 (22%)	3 (9%)
South Asian British	1 (5%)	2 (14%)	3 (9%)
South Asian Other	2 (10%)	1 (7%)	3 (9%)
Other	2 (10%)	1 (7%)	3 (9%)
Black Other	1 (5%)	0 (0%)	1 (3%)
Maternal employment status			
Not in employment	10 (50%)	10 (71%)	20 (59%)
Full or part‐time employed (returned to work)	8 (40%)	3 (22%)	11 (32%)
Full or part‐time employed (maternity leave)	2 (10%)	1 (7%)	3 (9%)
Relationship status			
Single	9 (45%)	4 (29%)	13 (38%)
Married	9 (45%)	3 (22%)	12 (35%)
In an unmarried relationship	2 (10%)	7 (49%)	9 (27%)
Number of children			
1	8 (40%)	4 (29%)	12 (35%)
2	4 (20%)	8 (57%)	12 (35%)
≥3	8 (40%)	2 (14%)	10 (29%)
Age range of participating child			
6–10 months	14 (70%)	9 (64%)	23 (68%)
11–36 months	6 (30%)	5 (36%)	11 (32%)
Children's social services involvement			
Never	12 (60%)	7 (49%)	19 (55%)
Past only	4 (20%)	4 (29%)	8 (24%)
Current	4 (20%)	3 (22%)	7 (21%)
Maternal primary recorded diagnosis			
Major depressive or recurrent depressive disorder/episode	8 (40%)	6 (43%)	14 (41%)
Emotionally unstable personality disorder	6 (30%)	6 (43%)	12 (35%)
Adjustment disorder/other reactions to severe stress	2 (10%)	1 (7%)	3 (9%)
Anxiety disorder	1 (5%)	0 (0%)	1 (3%)
No diagnosis recorded	2 (10%)	0 (0%)	2 (5%)
Post‐traumatic stress disorder	0 (0%)	1 (7%)	1 (3%)
Other Mental and Behaviour Disorders associated with the Puerperium	1 (5%)	0 (0%)	1 (3%)
Maternal SCID‐PD primary classification^a^			
Borderline	13 (65%)	10 (72%)	23 (68%)
Avoidant	5 (25%)	0 (0%)	5 (15%)
Obsessive compulsive	2 (10%)	2 (14%)	4 (12%)
Paranoid	0 (0%)	2 (14%)	2 (5%)
Maternal history of sexual and/or physical violence trauma			
Any age	13 (65%)	11 (79%)	24 (71%)
In childhood	9 (45%)	8 (57%)	17 (50%)
Maternal complex PTSD disturbances in self‐organisation	13 (65%)	8 (57%)	12 (62%)
Maternal lifetime history of visits to emergency department for psychiatric reasons			
Yes	7 (35%)	8 (57%)	15 (44%)
No	11 (55%)	5 (36%)	16 (47%)
Missing information	2 (10%)	1 (7%)	3 (9%)
Maternal lifetime history of self‐harm			
Yes	13 (65%)	13 (93%)	26 (77%)
No	6 (30%)	0 (0%)	6 (18%)
Missing information	1 (5%)	1 (7%)	2 (5%)
	Mean (*SD*)	Mean (*SD*)	Mean (*SD*)
Edinburgh Post‐natal Depression scale	14.5(4.6)	10.5 (5.3)	12.9 (5.2)
SAS‐PD scale^b^	10.6 (4.0)	10.9(4.0)	10.7(4.0)

^a^ Structured Clinical Interview for DSM‐V Personality Disorders.

^b^ Standardized Assessment of Severity of Personality Disorder.

### Feasibility outcomes

#### Primary feasibility outcome

About 95% of mothers allocated to the intervention arm received at least one VIPP‐PMH session (19/20; 95% CI 75%–100%), whilst 70% completed all six sessions (14/20; 95% CI 46%–88%).

#### Secondary feasibility outcomes

Secondary feasibility outcomes are summarised in Table 2.

**TABLE 2:** Secondary feasibility outcomes

	*n*/*N*	%	95% confidence interval
Consent for researcher contact	57/75^a^	76%	65%–85%
Informed consent rate	39/51^b^	76%	63%–87%
Month 5 follow‐up rate	29/34	85%	69%–95%
Month 8 follow‐up rate	22/34	65%	47%–80%

^a^ The denominator is mothers whom clinicians deemed potentially eligible and gave information about the research.

^b^ The denominator is mothers who were contactable by the researchers and eligible to participate.

### Pre‐post and between‐groups effects

Pre‐post and between‐groups descriptive statistics, effect sizes, and confidence intervals at baseline, month 5 and month 8 are shown in Table 3.

**TABLE 3:** Pre‐post and between‐groups effect sizes

Outcome	Timepoint	VIPP‐PMH arm			Control arm			Between‐groups		
		*n*	Mean ± *SD*, Median [IQR] or *n* (%)	Change from baseline	*n*	Mean ± *SD*, Median [IQR] or *n* (%)	Change from baseline	Mean or % difference (95% CI)	Hedges g or risk ratio (95% CI)	Change from baseline mean or % difference
Observer‐rated										
EAS maternal non‐intrusiveness^a^										
Non‐intrusive	Baseline	20	6 (30%)		14	4 (29%)		1% (−30% to 32%)	1.05 (0.36–3.05)	
Intrusive			14 (70%)			10 (71%)				
Non‐intrusive	5 months	17	4 (24%)	−6%	11	3 (33%)	4%	−9% (−43% to 25%)	0.65 (0.20–2.06)	−10%
Intrusive			13 (76%)							
Non‐intrusive	8 months	11	4 (36%)	6%	9	3 (33%)	4%	3% (−39% to 45%)	1.09 (0.33–3.65)	2%
Intrusive			7 (64%)			6 (67%)				
EAS maternal sensitivity^a^										
Sensitive	Baseline	20	7 (35%)		14	7 (50%)		−15% (−49% to 19%)	0.70 (0.32–1.55)	
Inconsistent or in‐sensitive			13 (65%)			7 (50%)				
Sensitive	5 months	17	9 (53%)	18%	11	3 (27%)	−23%	16% (−19% to 51%)	1.94 (0.67–5.63)	41%
Inconsistent or in‐sensitive			8 (47%)			8 (73%)				
Sensitive	8 months	11	7 (64%)	29%	9	3 (33%)	−17%	31% (−11% to 73%)	1.91 (0.68–5.33)	46%
Inconsistent or in‐sensitive			4 (36%)			6 (67%)				
Self‐reported										
BITSEA child behaviour problems^b^										
At risk	5 months	18	7 (39%)	n/a	11	2 (18%)	n/a	21% (−11% to 53%)	n/a	n/a
Not at risk			11 (61%)			9 (82%)				
At risk	8 months	13	2 (15%)	n/a	9	2 (22%)	n/a	−7% (−40% to 26%)	n/a	n/a
Not at risk			11 (85%)			7 (78%)				
CORE‐OM maternal mental health^c^	Baseline	20	1.8 ± 0.5	–	13	1.3 ± 0.7	–	0.5 (0.07–0.93)	0.83 (0.11–1.56)	
	5 months	18	1.6 ± 0.7	−0.2 ± 0.6	11	1.5 ± 0.6	0.2 ± 1.0	0.10 (−0.42 to 0.62)	0.15 (−0.60 to 0.90)	−0.4
	8 months	13	1.6 ± 0.7	−0.3 ± 0.5	9	1.4 ± 0.6	0.1 ± 0.9	0.20 (−0.40 to 0.80)	0.29 (−0.56 to 1.14)	−0.4
PSOC parenting confidence^d^	Baseline	19	67 ± 10	–	13	74 ± 11	–	−7.0 (−14.65 to 0.65)	−0.66 (−1.38 to 0.07)	
	5 months	18	71 ± 11	4 ± 10	11	73 ± 12	1 ± 10	−2.0 (−10.94 to 6.94)	−0.17 (−0.92 to 0.58)	3
	8 months	13	69 ± 11	4 ± 10	9	69 ± 9	−2 ± 10	0 (−9.27 to 9.27)	0 (−0.85 to 0.85)	6
PSS parenting stress^e^	Baseline	20	40 ± 9	–	13	34 ± 9	–	6 (−0.54 to 12.54)	0.65 (−0.07 to 1.37)	
	5 months	18	40 ± 9	0 ± 7	11	35 ± 9	1 ± 7	5 (−2.07 to 12.07)	0.54 (−0.22 to 1.30)	0
	8 months	13	41 ± 11	−1 ± 7	9	41 ± 9	4 ± 6	0 (−9.27 to 9.27)	0 (−0.85 to 0.85)	−5
ITSCL child attachment and emotional functioning^f^	Baseline	20	0 [0, 2]	–	12	0 [0, 2]	–	0		
	5 months	16	1 [0, 3]	0 [0, 1]	11	0 [0, 2]	0 [−2, 0]	1		
	8 months	13	0 [0, 0]	0 [−2, 0]	9	0 [0, 2]	0 [−2, 2]	0		
ITSCL child self‐regulation^f^	Baseline	20	4 [3, 7]	–	13	2 [1, 4]	–	2		
	5 months	18	2 [0, 4]	−2 [−1, 0]	11	0 [0, 4]	−2 [−1, 0]	2		
	8 months	12	2 [0, 6]	−1 [−5, 2]	9	0 [0, 6]	−2 [−1, 0]	2		

^a^ Emotional Availability Scale.

^b^ Brief Infant Toddler Socioemotional Assessment.

^c^ Clinical Outcomes in Routine Evaluation Outcome Measure.

^d^ Parenting Sense of Competence scale.

^e^ Parental Stress scale.

^f^ Infant Toddler Symptom Checklist.

#### Maternal sensitivity

**FIGURE 2:**
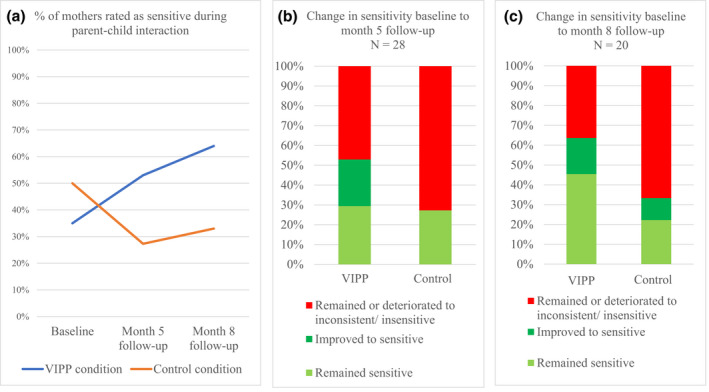
Maternal sensitivity during parent–child interaction. VIPP, Video feedback intervention for positive parenting

Blinded observer ratings of maternal sensitivity in parent–child interaction showed a substantial increase in the proportion of mothers rated as sensitive at month 5 and month 8 in the intervention condition, compared to a substantial decrease in the proportion of mothers rated as sensitive in the control condition, with twice as many mothers rated as sensitive in the intervention condition as in the control condition by the month 8 follow‐up (Figure 2a). Further analysis showed that this pattern was retained when examining complete cases and was unlikely to be due to differential drop‐out of sensitive mothers in the intervention versus control conditions (Supporting Information). Examining individual changes in sensitivity classification, mothers in the intervention condition were more likely to remain sensitive or improve to become sensitive between baseline and follow‐up, compared to mothers in the control condition, whereas mothers in the control condition were more likely to either remain insensitive or deteriorate to become insensitive between baseline and follow‐up, compared to mothers in the intervention condition (Figure 2b,c).

#### Maternal non‐intrusiveness

Blinded observer ratings of maternal non‐intrusiveness showed little change over time in the proportion of mothers rated as non‐intrusive, in either condition. There was a small difference in non‐intrusiveness at month 5 favouring the control condition.

#### Parenting confidence and parenting stress

**FIGURE 3:**
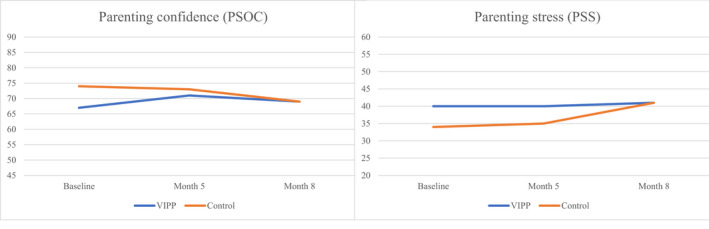
Parenting confidence and stress. PSOC, Parenting Sense of Competence scale; PSS, Parental Stress Scale; VIPP, Video feedback intervention for positive parenting

Between baseline and the follow‐up points, parenting confidence increased in the intervention group and decreased in the control group. Similarly, parenting stress remained stable over time in the intervention group and increased over time in the control group (Figure 3).

#### Child behaviour problems

**FIGURE 4:**
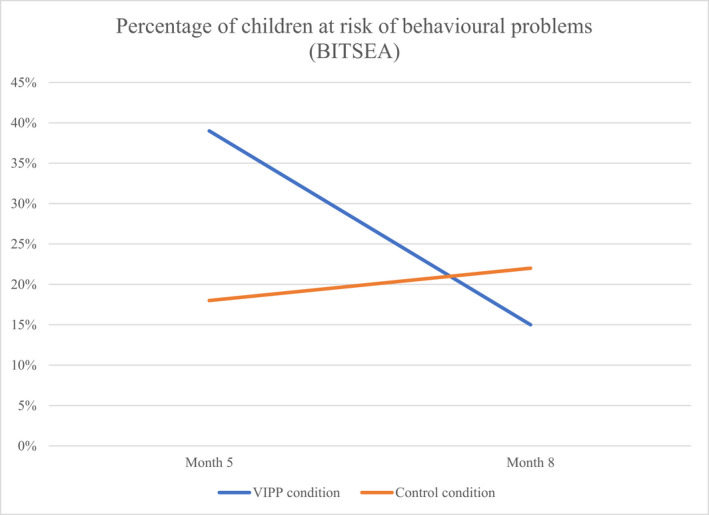
Child behaviour problems (BITSEA)

Child behaviour problems could not be rated at baseline as the majority of children were aged under 11 months. According to maternal ratings, at the month 5 follow‐up, more children were at risk of behaviour problems in the intervention than in the control group, and at the 8‐month follow‐up, more children were at risk in the control group than in the intervention group (Figure 4). Two children in the intervention condition lost their “at‐risk” status between month 5 and month 8, versus no children in the control condition.

#### Child attachment, emotional functioning and self‐regulation

Maternal ratings of child attachment and emotional functioning did not clearly change over time in either condition; maternal ratings of child self‐regulation improved over time in both conditions with no between‐groups differences.

#### Maternal mental health

Maternal mental health did not clearly change over time in either condition.

## DISCUSSION

This is, to our knowledge, the first published randomised controlled trial of a parent–infant intervention for parents experiencing enduring difficulties in managing emotions and relationships, consistent with a “personality disorder”. The majority of participating mothers had a history of sexual and/or violent trauma and exceeded the diagnostic threshold for disturbances in self‐organisation associated with complex PTSD, reinforcing the overlap between these diagnoses in clinical practice (Cloitre et al., 2014; Driessen et al., 2002; Frost et al., 2020; Giourou et al., 2018; Jowett et al., 2020).

### Feasibility and acceptability of the VIPP–PMH intervention and randomised controlled trial procedures

VIPP‐PMH intervention uptake and completion rates were high, indicating the acceptability of this approach for mothers experiencing enduring difficulties in managing emotions and relationships. Consent and post‐intervention follow‐up rates were also high, indicating the feasibility of the randomised controlled trial protocol. This aligns with a large body of international and UK evidence, showing that trials of original and adapted versions of VIPP have been feasible and acceptable in other at‐risk populations, including mothers with bulimia nervosa, insecure attachment or low sensitivity, and children with behavioural problems (Juffer et al., 2017; O'Farrelly et al., 2021). The VIPP model aims to help parents engage with and enjoy the intervention, through its emphasis on highlighting positive aspects of parent‐child interaction and celebrating parent strengths (Juffer et al., 2015). Our adaptation of the intervention sought to build on this to further increase acceptability for this group of parents by helping parents to manage any self‐critical thoughts or worries about being judged that may arise during the intervention. Post‐intervention follow‐up rates were high, but loss‐to‐follow‐up was higher at the final assessment point. This final follow‐up was affected by the COVID‐19 pandemic, which started at the point when some 8‐month follow‐ups were planned. Another contributing factor may have been that some final follow‐ups coincided with two research personnel changes.

### Potential effects of the VIPP‐PMH intervention

Parental sensitivity is the main target of the VIPP intervention due to its strong positive association with secure parent–child attachment and its link with a range of positive child socioemotional outcomes including reduced risk of child behaviour problems and mental health difficulties, and improved emotional, social, and academic functioning (Alink et al., 2009; De Wolff & van Ijzendoorn, 1997; Frick et al., 2018; Jaekel et al., 2015; Kok et al., 2013). Forty‐one percent of mothers entered our study exhibiting high levels of sensitivity in interacting with their children. This is important since mothers with diagnosed “personality disorder” say that this label can lead to professionals making negative assumptions about their parental capacity (Zacharia et al., 2021). We found potential evidence of a large positive within and between‐groups effect of VIPP‐PMH on maternal sensitivity during parent–child interaction. In the intervention condition, there was a substantial increase between baseline and each follow‐up in the numbers of mothers rated by a blinded psychologist as sensitive during mother–child interaction. By contrast, in the control condition, there was a substantial decrease over time in the numbers of mothers rated as sensitive. The intervention group mothers were twice as likely as the control group mothers to be rated as sensitive at either follow‐up point. Due to the small sample size, statistical significance could not be determined and hence it is difficult to be certain whether these within‐group changes and between‐groups differences are attributable to the intervention or occurred by chance. However, if the effects are attributable to VIPP‐PMH, they suggest the intervention may not only help promote improvements in maternal sensitivity for mothers who have difficulties in this area but may also reinforce positive interactive behaviour in mothers who enter the intervention with high levels of sensitivity, acting as a protective factor against the decline in sensitivity that may otherwise occur as babies enter the emotionally turbulent and behaviourally challenging phase of toddlerhood (Bridgett et al., 2009; Lipscomb et al., 2011; Partridge & Lerner, 2007). This highlights the importance of early intervention (Heckman & Masterov, 2007), whereby offering mothers support in their child's early infancy could prevent later difficulties from developing.

We also found a potential small positive effect of VIPP‐PMH on parenting confidence and parenting stress. Elements of VIPP that may help with these areas are its emphasis on highlighting positive aspects of parent–child interaction and celebrating parents' strengths, normalising the child's behaviour, and empathising with both parent and child (Juffer et al., 2015). Taken together, these findings indicate that further large‐scale studies are warranted with this population to explore the potential positive benefits of VIPP‐PMH for mothers and their young infants.

By contrast, there were no clear intervention effects on observer ratings of maternal non‐intrusiveness or maternal ratings of their own mental health or of child behaviour problems, attachment, and emotional functioning or self‐regulation. The lack of positive effect on maternal non‐intrusiveness is contradictory to research in other populations, including families experiencing poverty, families with an autistic child, and ethnic minority families, which showed benefits of VIPP over usual care for improving non‐intrusiveness (Negrão et al., 2014; Poslawsky et al., 2015; Yagmur et al., 2014). This suggests that intrusive behaviour may be harder to change than sensitivity in our study population. Pejorative explanations have been given for why women with a “BPD” diagnosis may sometimes seem intrusive when interacting with their children, including references to their relationships with others as “intense and disturbed”, and their difficulties in being alone (Biringen, 2008; Crandell et al., 2003). We offer a more benign interpretation. In other research, mothers with “BPD” or complex trauma describe strong motivation to parent positively, whilst lacking a template for how to do so (Dunn et al., 2020; Siverns & Morgan, 2019). Practitioners have highlighted that some mothers cope with their own feelings of uncertainty and self‐doubt by exerting control over parent–child interaction (Dunn et al., 2020), whilst mothers with a “BPD” diagnosis have described gaining self‐worth from teaching and moulding their child (Bartsch et al., 2016). This may have led to some parents in our sample over‐involving themselves in their child's play, enthusiastically teaching them how to use the toys and directing their activities, rather than stepping back and letting their child lead.

### Limitations

The small sample size increases the risk of differences between intervention and control participants occurring due to chance. Follow‐up rates decreased at longer‐term follow‐up. Baseline child behaviour problems could not be assessed due to the young age of most participating children. Generalisability of our findings to fathers cannot be assumed.

### Implications for clinical practice and further research

Our research shows that the video feedback intervention for positive parenting adapted for perinatal mental health difficulties (VIPP‐PMH) is feasible and acceptable to implement with women experiencing enduring difficulties in managing emotions and relationships consistent with a “personality disorder” in the perinatal period. Our findings also highlight potential benefits of the intervention for reinforcing or improving maternal sensitivity in parent–child interaction and improving parenting confidence and stress. Furthermore, they highlight the strong potential of this group of mothers for resilience, growth, and positive parent–child relationships. Further intervention optimisation, such as more explicit psychoeducation for parents about the benefits of stepping back and letting their child lead the interaction, may better support improvements in non‐intrusiveness. Additionally, VIPP‐PMH is a brief six‐session intervention. Brief interventions have been shown meta‐analytically to be more effective than longer interventions for improving maternal sensitivity and infant attachment security (Bakermans‐Kranenburg et al., 2003). However, to improve intervention effects on maternal mental health, it may be useful to investigate the effectiveness of integrating or supplementing VIPP‐PMH with longer‐term interventions focussed on improving maternal emotion regulation, such as Mother‐Infant Dialectical Behaviour Therapy (Sved Williams et al., 2021) or the Parenting Skills for Borderline Personality Disorder Group Training (Renneberg & Rosenbach, 2016), potentially using a stepped care model.

Having met our pre‐specified progression criteria, our findings reinforce the value of conducting a definitive trial of VIPP‐PMH in this group of parents and their children. The trial should be powered to detect minimum clinically important differences between study arms. Additionally, longer‐term outcomes should be assessed. Loss to follow‐up should be reduced by maintaining consistent participant–researcher relationships and keeping in touch in‐between assessments.

Finally, the difficulties in recruiting fathers were striking. Clinical teams seemed less used to thinking of men with this set of difficulties as “fathers”, and, commensurate with previous research, those that were identified had little contact with their children (Lumsden et al., 2018). This brings into focus how little research and clinical attention is given to fathers experiencing severe mental illness (Price‐Robertson 2015). Further research should use father‐targeted recruitment materials to better engage fathers (Yaremych & Persky, 2022), should investigate the impact of paternal severe mental illness on parent–child relationships, and should explore how fathers can better be identified and supported.

## AUTHOR CONTRIBUTIONS

**Kirsten Barnicot:** Conceptualization; data curation; formal analysis; funding acquisition; investigation; methodology; project administration; supervision; writing – original draft; writing – review and editing. **Morgan Welsh:** Formal analysis; writing – review and editing. **Sarah Kalwarowsky:** Data curation; investigation; project administration; writing – review and editing. **Eloise Stevens:** Methodology; supervision; writing – review and editing. **Jane Iles:** Conceptualization; funding acquisition; supervision; writing – review and editing. **Jennie Parker:** Conceptualization; data curation; funding acquisition; investigation; writing – review and editing. **Maddalena Miele:** Project administration; supervision. **Tara Lawn:** Project administration; supervision. **Laura O’Hanlon:** Project administration; resources. **Sushma Sundaresh:** Project administration; supervision. **Awawu Ajala:** Project administration. **Paul Bassett:** Formal analysis; funding acquisition. **Christina Jones:** Supervision; writing – review and editing. **Paul Ramchandani:** Conceptualization; funding acquisition; supervision; writing – review and editing. **Mike Crawford:** Conceptualization; funding acquisition; supervision; writing – review and editing.

## CONFLICT OF INTEREST

All authors declare no conflicts of interest.

### OPEN RESEARCH BADGES

This article has earned a Preregistered Research Designs badge for having a preregistered research design, available at https://www.isrctn.com/ISRCTN10052006.

## Supporting information


Appendix S1

## Data Availability

The data that support the findings of this study are available on request from the corresponding author. The data are not publicly available due to privacy and ethical restrictions as they contain information that could compromise the privacy of research participants, and participants have not consented to data publication.

## Footnotes

^1^
Participants were all biologically female to our knowledge; however, gender identity was not assessed.
