# Supplementary material for: Video feedback parent‐infant intervention for mothers experiencing enduring difficulties in managing emotions and relationships: A randomised controlled feasibility trial
Source: Br J Clin Psychol. 2022 Aug 26;61(4):1188–210. doi: 10.1111/bjc.12388 (PMC9804867; doi:10.1111/bjc.12388)
Supplement: Supplementary file 1 — Appendix S1 [file BJC-61-1188-s001.docx]

| Question  Sociodemographics, Treatment History and Risk Interview | Answer |
| --- | --- |
| 1. What is your date of birth? [DD/MM/YY] |  |
| 2. Male or female? |  |
| 3. How would you describe your ethnicity? [Please circle] | White British  White Other  Black British  Black Other  British South Asian  Other South Asian  British East Asian  Other East Asian  Mixed Black + White  Mixed Black + South Asian  Mixed Black + East Asian  Mixed White + South Asian  Mixed White + East Asian  Other [Please specify] |
| 4. Are you currently employed? [Please circle] | Full time Part time Unemployed |
| 5. Are you currently studying? [Please circle] | Full time Part time Not studying |
| 6. What was your job title in your most recent job? |  |
| 7. What is your highest educational qualification? |  |
| 8. How many children do you have and what are their ages? | Child 1 Age  Child 2 Age  Child 3 Age  Child 4 Age  Child 5 Age |
| 9. What is your current relationship status? | Married In a relationship Single |
| 10. Are you currently in a relationship with the other parent of the child taking part in this research? | Yes No |
| 11. What psychiatric medications are you currently prescribed? |  |
| 12. Thinking back, what year was it or how old were you when you were you first referred to psychiatric services? |  |
| 13. Have you ever got to a point where you were struggling to cope with your mental health to the point where you wentto A&E? [If yes] How many times in your life? |  |
| 14. Have you ever been admitted to an inpatient psychiatric hospital? [If yes] How many times? |  |
| 15. Have you ever got to a point where you’ve felt so distressed that you’ve lashed out at yourself and deliberately hurt yourself? [If yes].. In your whole life, has that happened 4 or more times? [etc] | 0 = no  1 = yes, once  2 = yes, 2-3 times  3 = 4 or more times |
| 16. [If yes to Q 15], On any of those times, did you ever have to go toto a general hospital because of the physical damage caused by self-harm?  [If yes] Did you need to stay overnight?  [If yes] Did you need to go to the intensive care unit, or did you suffer lasting physical damage? | 0 = no, never self-harmed  1= yes, physical effects did not require admission to general hospital  2= yes, required admission at least overnight to general hospital, but no lasting effects and no admission to intensive care unit  3 = self-harm is likely to have caused lasting physical damage (other than superficial scarring) and/or required admission to intensive care unit |
| 17. [If yes to Q 15,] in the last 12 months have you deliberately hurt yourself? | 0 = no  1 = yes, once  2 = yes, 2-3 times  3 = 4 or more times |
| 18. [If yes to Q 17], On any of those times in the last 12 months, did you ever to go to a general hospital because of the physical damage caused by self-harm?  [If yes] Did you need to stay overnight?  [If yes] Did you need to go to the intensive care unit, or did you suffer lasting physical damage? | 0 = no, never self-harmed  1= yes, physical effects did not require admission to general hospital  2= yes, required admission at least overnight to general hospital, but no lasting effects and no admission to intensive care unit  3 = self-harm is likely to have caused lasting physical damage (other than superficial scarring) and/or required admission to intensive care unit |
| 19. Have you ever got a to a point where you’ve felt so angry and worked up that you’ve lashed out at someone else and maybe assaultedthem , committed a sexual offence, or threatened them with violence? [If yes] In your whole life, has that happened 4 or more times? [etc] | 0 = no  1 = yes, once  2 = yes, 2 or 3 times  3 = yes, 4 or more times |
| 20. [If yes to Q. 19], thinking of the most serious occasion on which this happened[Ask whichever seems applicable based on their answers]:  Did it ever turn into a physical assault or was it just threats?  Did you have a weapon?  Did the other person need to go to hospital?  Etc. | 0 = nil  1 = no assault, but minor verbal aggression  2 = no assault, but has made repeated threats to inflict significant harm on someone or has threatened someone with a weapon  3 = assault not resulting in any need for victim to have hospital in-patient treatment  4 = assault resulting in victim needing hospital in-patient treatment, but not in lasting disability assault resulting in lasting disability homicide  5 = assault resulting in lasting disability  6 = homicide |
| 21. [If yes to Q. 19],And in the past 12 months has it happened where you’ve got so worked up that you’ve assaulted someone or threatened violence? If yes] In the past 12 months, has that happened 4 or more times? [etc] | 0 = no  1 = yes, once  2 = yes, 2 or 3 times  3 = yes, 4 or more times |
| 22. [If yes to Q. 21], thinking of the most serious occasion on which this happened in the past 12 months….  [Ask whichever seems applicable based on their answers]:  Did it ever turn into a physical assault or was it just threats?  Did you have a weapon?  Did the other person need to go to hospital?  Etc. | 0 = nil  1 = no assault, but minor verbal aggression  2 = no assault, but has made repeated threats to inflict significant harm on someone or has threatened someone with a weapon  3 = assault not resulting in any need for victim to have hospital in-patient treatment  4 = assault resulting in victim needing hospital in-patient treatment, but not in lasting disability assault resulting in lasting disability homicide  5 = assault resulting in lasting disability  6 = homicide |
| 23. [NB you don’t need to ask this if you know the answer already!] Were you seen by perinatal mental health when you were pregnant or after you gave birth to [study child]?  [If applicable] What about when you were pregnant with [other children]? | 0 = no perinatal mental health involvement with any children  1 = perinatal mental health involvement only with study child  2= perinatal mental health involvement only with other children  3 = perinatal mental health involvement with both study child and other children |
| 24. Have you ever had children’s social services involved with [study child]?  [If yes] Are they currently involved or was that in the past?  [If applicable] Have you ever had children’s social services involved with [other children]?  [If yes] Are they currently involved or was that in the past? | NB you can circle more than one here!  0 = no children’s social services involvement with study child  1 = past children’s social services involvement with study child  2 = current children’s social service involvement with study child 3 = current children’s social services involvement with other children  4 = past children’s social services involvement with other children |

Online Supplementary Table 1. Usual Care Received

|  |  | Months 1 to 5 number of mothers receiving care  n (%) | | Months 6 to 8 number of mothers receiving care  n(%) | |
| --- | --- | --- | --- | --- | --- |
| Service | Profession/ role | VIPP arm  N = 18 | Control arm  N = 11 | VIPP arm  N = 13 | Control arm  N = 9 |
| Perinatal mental health service | Psychiatrist | 4 (22%) | 0(0%) | 0(0%) | 2 (22%) |
|  | Psychologist/ psychotherapist | 5 (28%) | 4 (36%) | 1 (8%) | 2 (22%) |
|  | Nurse/ mental health social worker/ other | 4 (22%) | 1 (9%) | 0(0%) | 0(0%) |
| Other mental health service | Psychiatrist | 1 (6%) | 0(0%) | 2 (15%) | 0(0%) |
|  | Psychologist/ psychotherapist | 3 (17%) | 1 (9%) | 2 (15%) | 1 (11%) |
|  | Nurse/ mental health social worker/ other | 2 (11%) | 1 (9%) | 0 (0%) | 1 (11%) |
| Privately funded | Psychologist/ psychotherapist | 2(11%) | 1 (9%) | 1(8%) | 0(0%) |
| GP | Discuss maternal mental health | 6 (33%) | 6(55%) | 4(31%) | 2 (22%) |
|  | Discuss child mental health/ development | 2 (11%) | 0(0%) | 1 (8%) | 1 (11%) |
| Child services | Health visitor | 10 (56%) | 8 (73%) | 4 (31%) | 2 (22%) |
|  | Children’s social worker/ family support worker | 5 (28%) | 2 (18%) | 2 (15%) | 3 (33%) |
|  | Other child health professional † | 7(39%) | 2 (18%) | 2 (15%) | 1 (11%) |

† Including allergy clinic, child psychologist, children’s centre staff, dietician, Family Matters charity, paediatrician, parenting class, physiotherapist, speech and language therapist, Sure Start

Online Supplementary Table 2. Assessment completion rates by baseline sensitivity level and trial arm

|  | Mothers rated as sensitive at baseline | | | |
| --- | --- | --- | --- | --- |
|  | Completed month 5 assessment | Did not complete month 5 assessment | Completed month 8 assessment | Did not complete month 8 assessment |
| VIPP | 6 | 1 | 5 | 2 |
| Control | 5 | 2 | 4 | 3 |
| Mothers rated as inconsistent/ insensitive at baseline | | | | |
| VIPP | 11 | 2 | 6 | 7 |
| Control | 6 | 1 | 5 | 2 |

Online Supplementary Figure 1. Sensitivity ratings for mothers completing the month 5 follow-up

Online Supplementary Figure 2. Sensitivity ratings for mothers completing the month 8 follow-up
